# The Topological Characteristics of Biological Ratio-Sensing Networks

**DOI:** 10.3390/life13020351

**Published:** 2023-01-28

**Authors:** Xinmao Chen, Tianze Wang, Ying Guan, Qi Ouyang, Chunbo Lou, Long Qian

**Affiliations:** 1 School of Physics, Peking University, Beijing 100871, China; 2 Center for Quantitative Biology, Peking University, Beijing 100871, China; 3 Center for Cell and Gene Circuit Design, CAS Key Laboratory of Quantitative Engineering Biology, Guangdong Provincial Key Laboratory of Synthetic Genomics, Shenzhen Key Laboratory of Synthetic Genomics, Shenzhen Institute of Synthetic Biology, Shenzhen Institutes of Advanced Technology, Chinese Academy of Sciences, Shenzhen 518055, China

**Keywords:** ratio sensing, design principle, reverse engineering, synthetic biology

## Abstract

Ratio sensing is a fundamental biological function observed in signal transduction and decision making. In the synthetic biology context, ratio sensing presents one of the elementary functions for cellular multi-signal computation. To investigate the mechanism of the ratio-sensing behavior, we explored the topological characteristics of biological ratio-sensing networks. With exhaustive enumeration of three-node enzymatic and transcriptional regulatory networks, we found that robust ratio sensing was highly dependent on network structure rather than network complexity. Specifically, a set of seven minimal core topological structures and four motifs were deduced to be capable of robust ratio sensing. Further investigations on the evolutionary space of robust ratio-sensing networks revealed highly clustered domains surrounding the core motifs which suggested their evolutionary plausibility. Our study revealed the network topological design principles of ratio-sensing behavior and provided a design scheme for constructing regulatory circuits with ratio-sensing behavior in synthetic biology.

## 1. Introduction

**Figure 1:**
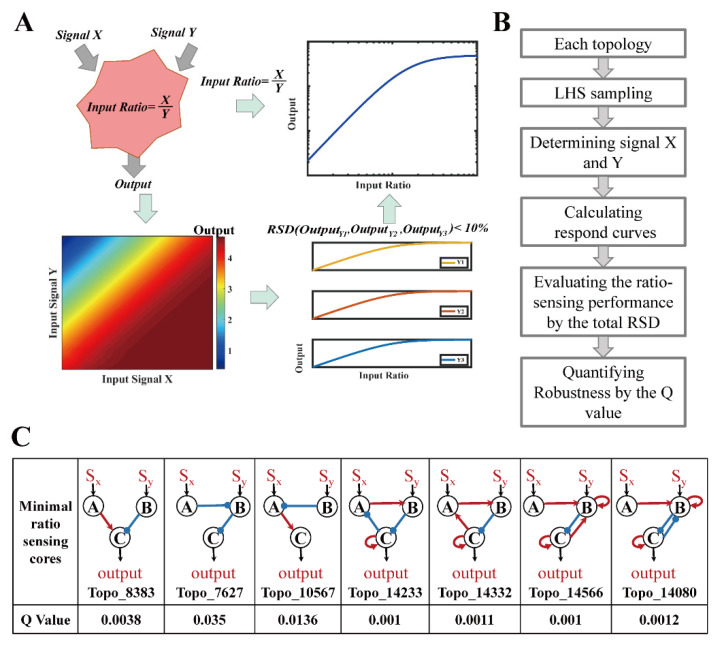
Definition of ratio sensing and the work flow of three-node EN enumeration. (**A**) Schematic of defining and measuring the ratio-sensing behavior in a two-input-one-output network configuration. If and only if the total relative standard deviation (RSD) across all stimulatory ratios was less than 10%, we considered the topological structure with a certain parameter set capable of ratio-sensing; (**B**) The work flow of computational enumeration of three-node networks. Each topology was independently simulated the input–output response functions for three different concentrations of Y1, Y2, Y3, and the ratio range (0.01~10) with 10^4^ parameter sets with the Latin hypercube sampling (LHS) method. Then, the total RSD across all stimulatory ratios was measured, and if and only if it was less than 10%, the topology with the certain parameter set was capable of ratio sensing. The robustness for each topology was quantified by the Q-value, which was the fraction of parameter sets with which the topology exhibited ratio-sensing behavior; (**C**) Seven minimal ratio-sensing core topologies for three-node enzymatic networks (ENs) and their respective robustness. After topology pruning to remove redundant edges, each regulatory edge in the minimal core topologies was necessary for the Q-value to stay above the 0.001 cutoff. For each topology, black arrows represented the activation of nodes by external stimulatory signals, red arrows represented activation, and blue blunt arrows represented inhibition.

In fundamental living processes such as growth, development, and metabolism, cells use complex regulatory networks to integrate and respond to multiple signals for decision making. Upon the discovery of the carbon catabolite repression [1,2,3,4], it was found that the expression of the master gene GAL was induced at a specific ratio of external galactose and glucose concentrations despite the fact that the actual concentrations were varied across a wide range [5,6,7]. In signal transduction networks, this manner of regulation is called ratio sensing, i.e., cells respond to the ratio of two signals regardless of their absolute intensities (Figure 1A). Besides the glucose metabolism pathway, ratio sensing was later discovered in circadian rhythms, apoptosis, and other pathways. Different ratios of the circadian regulator proteins Cry1 and Cry2 could induce circadian oscillations in a cell-cycle-dependent manner [8]. The apoptosis program was influenced by the ratio of Bax to Bcl-2, which serves as a predictor of clinical drug resistance and the apoptotic potential of cancer cells [9]. The ratio of ATP to ADP was also an important indicator in a broad range of cellular programs [10,11,12]. Computational studies suggested natural signal transduction pathways could generate ratio-sensing behaviors. For example, by exploring the parameter space, Antebi et al. showed that the BMP signaling pathway could generate a ratio-sensing response through differential ligand-receptor affinities [13]. On the other hand, synthetic regulatory circuits were constructed in *Escherichia coli* and yeast cells to perform ratio sensing as a demonstration of cellular analog computing. These circuits were implemented with competitive transcription factors [14,15] or RNA-guided dCas9 transcriptional regulators [16].

Network motifs are small recurring patterns of interactions within a large network [17]. For several extensively studied regulatory networks, motifs present an epitome for network functionality [18]. Enumeration of coarse-grained three-node networks revealed the topological essentialities for network functions including adaptation [19], drosophila segment polarity [20], dose-response aligned circuits [21], self-organizing cell polarization [22], ultrasensitive quorum-sensing switch [23], Turing pattern [24], Pavlovian-like associative memory [25], temperature insensitive oscillation [26], oscillation with incoherent inputs [27,28], multiple signal-encoding functions [29], biochemical timers [30], etc. In some of the abovementioned studies, the deduced motifs were used for reverse engineering synthetic circuits to reproduce the biological functions [23,31]. The work flow of enumeration of three-node networks consisted of three steps. Firstly, the total enumerated three-node topological candidates should be determined. Secondly, each three-node topology was simulated independently for the input–output response functions with 10,000 parameter sets obtained by the Latin hypercube sampling (LHS) method (Figure 1B). The robustness for each three-node topology was quantified by the Q-value. Thirdly, statistical analysis of the total enumerated three-node topological candidates with their Q-values was conducted to explore the relationship of the function and structures for the enumerated three-node topological candidates.

Here, by taking a similar approach, we explored the network topological characteristics and the mechanism of ratio-sensing response function. We first enumerated three-node enzymatic networks to search a space of around 18,000 possible network topologies, and found seven minimal core topological structures and four motifs for ratio sensing. Of the four motifs, three gave rise to most of network topological structures with high robustness. Then, the origin of robust ratio sensing for one of the core motifs was derived analytically. By tracing robustness along the mutational paths in the evolution space of all topological structures, we found strong connectivity between the three motifs, while the fourth motif was likely to have evolved independently. Finally, we applied the analysis to transcriptional networks to arrive at three minimal core topological structures and three motifs. These analyses revealed a universal design principle for biological ratio-sensing networks.

## 2. Materials and Methods

### 2.1. The Coarse-Grained Computational Enumeration of 3-Node Networks

To identify the network topological characteristics of ratio-sensing behavior, we enumerated all topologies for three-node enzymatic networks (EN) and transcriptional regulatory networks (TRNs), as described previously [19,32]. For each topology, there were three nodes and totally nine possible regulatory relationships between three nodes. A three-dimensional adjacency matrix *J* was used to describe the regulatory relationships between nodes, in which each row represented a node and each matrix element *J_ij_* represented a possible regulatory relationship. The matrix element *J_ij_* could be 1, −1, or 0 which represented: node *i* (*i* = A, B, or C) was activated (1), inhibited (−1), or no regulatory relationship (0) by node *j* (*j* = A, B or C), respectively. Therefore, the topological structure was described by the three-dimensional adjacency matrix *J.* In addition, each topology had a unique topological ID according to its three-dimensional adjacency matrix *J.* For example, a topology consisting of an activation edge from node A to C and an inhibition edge from node B to C was named as Topo_8383 and represented by the three-dimensional adjacency matrix $\;J=\left[\begin{array}{ccc} 0 & 0 & 0\\ 0 & 0 & 0\\ 1 & -1 & 0 \end{array}\right].$ Similarly, Topo_7627 with $J=\left[\begin{array}{ccc} 0 & 0 & 0\\ -1 & 0 & 0\\ 0 & -1 & 0 \end{array}\right]$ represented a topology consisting of an inhibition edge from node A to B and an inhibition edge from node B to C.

There were 3^9^ = 19,683 topological candidates for the coarse-grained computational enumeration. However, combined with experimental experience from synthetic biology and the assumption that nodes A and B received external stimulatory signals X and Y and the node C was the output node, the topologies in which the dual input stimulatory signals were not transduced to output node C were discarded. Thus, there were only total 17,496 enumerated three-node topological candidates.

For three-node ENs, each node in the network represented a protein having a fixed total concentration (normalized to 1) for active and inactive forms that interconverted, and every directed line segment between nodes represented their regulatory relationship, i.e., activation or inhibition. For three-node TRNs, each node represented a transcription factor regulated by other nodes.

### 2.2. Modeling Network Behavior with Ordinary Differential Equations

For three-node ENs, the Michaelis–Menten equation was used to model the process that stimulatory signals X and Y transformed protein A and B from inactive states to active states through enzymatic regulations, respectively, followed by the enzymatic interactions between proteins A, B, and C and their basal enzymes, as described previously [19]. The total concentrations of nodes A, B, and C were normalized to 1. If a node *i* (*i* = A, B, or C) was not inhibited by any other node, a basal enzyme *F_i_* was assumed to inactivate that node from its active to inactive form with the Michaelis–Menten constants $K_{MF_{i}}$. Specifically, for every topology, nodes A and B received external stimulatory signals X and Y, respectively, and the node C was detected as the output signal. The dynamic behavior of the whole network was determined by the ordinary differential equations of concentrations of all nodes. The parameters of the Michaelis–Menten constants $K_{Mi}$ for node *i* were in the range of (10^−2^ a.u.~10^2^ a.u.) and the parameters of catalytic rate constants k of the enzymes were in the range of (10^−1^ a.u.~10^1^ a.u.), and the basal enzymes *F_i_* of node *i* of nodes were 0.01 as described previously [27].

For three-node TRNs, the general transcriptional regulatory model [32] with transfer functions of dual input stimulatory signals was built for the process that the two transcription factors A and B which regulated the output C production were activated by the stimulatory signals X and Y, respectively. In addition, the parameter $\gamma_{i}$ for node *i* was the rate of degradation and dilution. The Hill coefficients were in the range of (1~3) from reference [33], and the parameters of the binding affinities $K_{i}$ were in the range of (10^−1^ a.u.~10^3^ a.u.) from reference [34,35]. Considering the overexpression of the transcription factor in experiments and the derivation of the equation where the maximum expression rates did not significant limit the function of ratio sensing, we assumed the parameters of the maximal transcriptional rates $\alpha_{i}$ were in the range of (10^3^ a.u.~10^6^ a.u.).

Latin hypercube sampling (LHS) method is a method of approximating random sampling from multivariate parameter distribution, which belongs to hierarchical sampling technology. Every sample obtained by LHS is of equal probability in all parameters, which is commonly used in computer experiments. Compared with Monte Carlo random sampling where parameters would cluster in the high-dimensional parameter space, LHS is able to uniformly cover the entire parameter space with a smaller number of samples. These parameters were generated at the logarithmic scale by the Latin hypercube sampling method. All computational enumerations and simulations were executed by Dev C++ and Polaris High Performance Computing platform.

### 2.3. Definition and Quantification of Ratio-Sensing Performance

Ratio sensing is defined by an output response remaining constant when the intensities of the dual input stimulatory signals change in proportion. For a given network, output response curves can be plotted as a function of the ratio between two inputs when the absolute magnitudes of inputs change. Response curves overlaying with each other indicates ratio sensing (Figure 1A). In practice, the relative standard deviation (RSD) between response curves could be used as a measure, and topologies with RSD below 10% were defined as ratio-sensing.

Specifically, to determine the robustness for each network topology, we used LHS method to generate 10,000 parameter sets and independently simulated the input–output response functions (Figure 1B), which roughly consisted of four steps. Firstly, we determined the parameters in the model with one set of the 10,000 parameter sets and the concentration range of signal X corresponding to three different concentrations of Y (Y1 = 5 a.u., Y2 = 100 a.u., and Y3 = 500 a.u.) within the ratio range of (0.01~10). Secondly, we calculated the three response curves with determined signal X and signal Y (Figure 1A). Thirdly, we evaluated the ratio-sensing performance by calculating the RSD between three response curves at sampled X/Y ratios. If and only if the total RSD across all stimulatory ratios was less than 10%, we considered the topological structure with a certain parameter set capable of ratio sensing. Lastly, the robustness for each network topology was quantified by the Q-value within the range of (0~1) (Figure 1B). Q-value was defined as the fraction of parameter sets with which the topology exhibited ratio-sensing behavior, which was a measure of the number of parameter sets satisfying the ratio-sensing criteria (RSD < 10%) relative to the 10,000 parameter sets. For example, the EN Topo_8383 with 38 parameter sets capable of ratio sensing had a Q-value 0.0038. Obviously, the higher the Q-value was, the more likely it was for the topology to display ratio-sensing behavior, and the less sensitive the behavior was toward parameter perturbations. Topologies with Q-value > 0.001 were regarded viable [27].

### 2.4. The Minimal Core Topologies and Motif Selection

According to previous work [30,36], the minimal core topology was defined as a topology in which each regulatory edge was necessary for the Q-value staying above the 0.001 cutoff, i.e., removing any edge from the topology would result in a Q-value below the 0.001 cutoff. We performed topology pruning with the 0.001 cutoff to remove redundant edges and found the minimal core topologies (Figure 1C and Figure 3A) that exhibited robust ratio sensing with the Software MATLAB R2018a.

**Figure 2:**
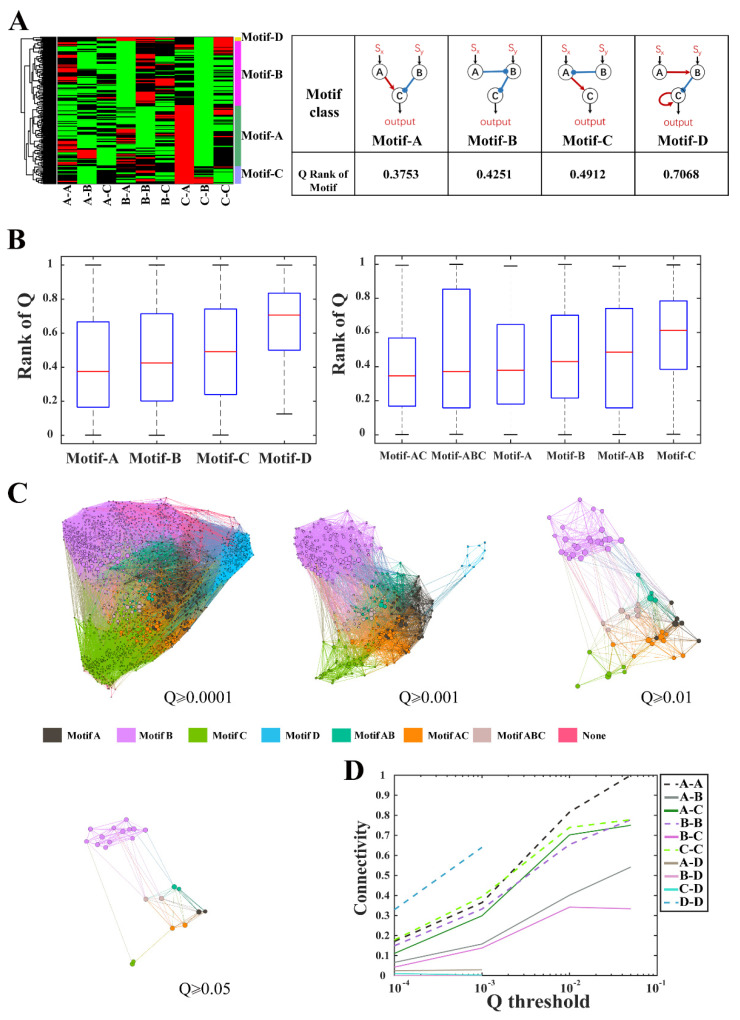
Structural characteristics of ratio-sensing topologies for three-node ENs. (**A**) Four classes of core ratio-sensing motifs. Each row in the cluster diagram represented a network topology from all 374 topologies of Q ≥ 0.001, and each column represented the regulatory relationship between a pair of nodes (red—activation, green—inhibition, black—no interaction). Four core ratio-sensing motifs were listed in the table with the overall robustness of all topologies in the motif classes; (**B**) Rank of the robustness for the four motif classes and their hybrids. All topologies were ranked according to their Q-values in descending order. If a network topological structure was a combination of the motifs A and B, it was classified as motif AB; (**C**) Metagraphs showing the domains that the motif classes occupied in the evolutionary space with different robustness cutoffs of 0.0001, 0.001, 0.01, and 0.05. In a metagraph, each node represents a topology and the node size is proportional to the Q-value of the corresponding topology, and a link is drawn if two topologies can interconvert through a change in one edge. Topologies with Q-values smaller than the cutoffs were removed from the metagraphs; (**D**) The connectivity between motif classes at different robustness thresholds. The connectivity between two motif classes is defined as the number of linkages between nodes of the two motif classes normalized by the product of the numbers of nodes in each motif class.

**Figure 3:**
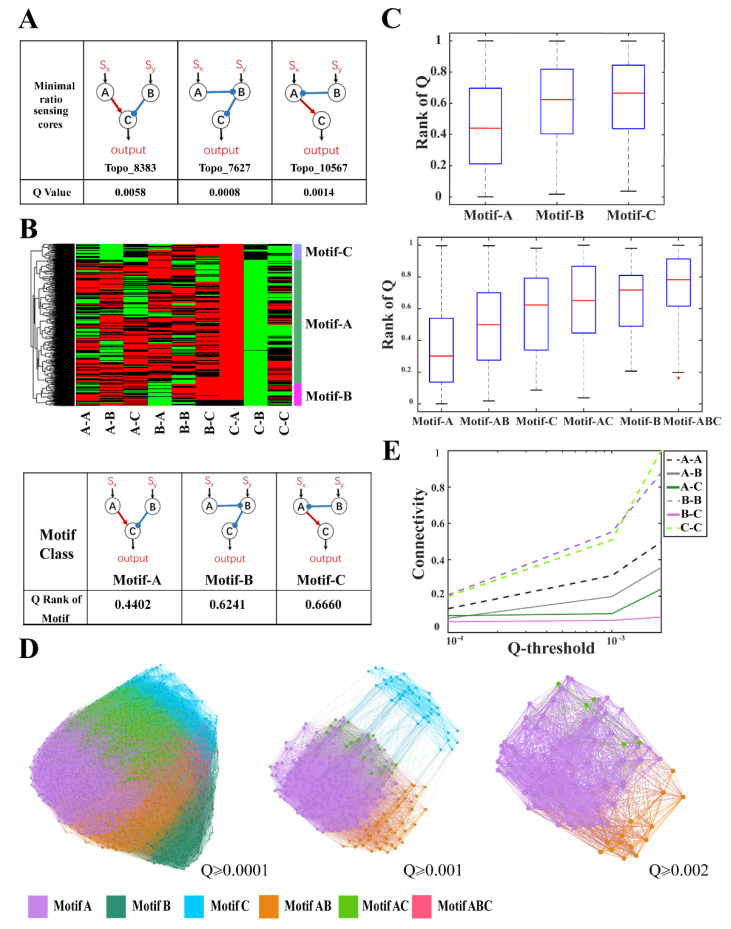
Structural characteristics of ratio-sensing topologies for three-node TRNs. (**A**) Three minimal ratio-sensing core topologies for three-node TRNs and their respective robustness. The Q-value threshold for topological pruning was 0.0005; (**B**) Cluster diagram of 384 TRN topologies (top, Q > 0.001) and the core motifs in three motif classes (bottom); (**C**) Boxplots of the rank of robustness for the three motif classes and their hybrids; (**D**) Metagraphs showing the domains that the motif classes occupied in the evolutionary space with different robustness cutoffs of 0.0001, 0.001, and 0.002. Topologies with Q-values smaller than the cutoffs were removed from the metagraphs; (**E**) The connectivity between motif classes with different robustness thresholds.

In addition, the cluster diagrams of the highly robust network topologies for motif classes (Figure 2A and Figure 3B) were obtained by the function ‘clustergram’ with Euclidean distance between the topology pairs by the MATLAB R2018a.

### 2.5. Evolutionary Metagraph

The evolutionary metagraph of robust ratio-sensing networks was built by analyzing the probability of one topology changing to another through changes in edges, which could be seen as “mutations”. In a metagraph, each node represents a topology, and the link weight is the reciprocal of the Euclidean distance between the topology pairs.

A visualization of the network was constructed using Gephi’s [37] built-in ForceAtlas2 [38] layout, a force-directed continuous layout algorithm. The default parameters of the algorithm were applied, including tolerance of 1.0, approximation of 1.2, scaling of 2.0, and gravity of 1.0. The node size is proportional to the Q-value of the corresponding topology using the circular spline, and the link width is linearly proportional to the link weight. Topologies with Q-values smaller than the cutoffs were removed from the metagraphs accordingly, after the metagraph with the loosest threshold was built first.

## 3. Results

### 3.1. Ratio-Sensing Definition and Three-Node Network Enumeration

Ratio sensing is defined by an output response remaining constant when the intensities of the dual input stimulatory signals change in proportion. In practice, the relative standard deviation (RSD) between response curves with different absolute intensities of the dual input stimulatory signals could be used as a measure, and topologies with total RSD below 10% were defined as ratio-sensing.

To identify the network topological characteristics of ratio-sensing behavior, we enumerated all topologies for three-node enzymatic networks (EN), as described previously [19]. Each node in the network represented a protein having a fixed total concentration for active and inactive forms that interconverted, and every directed line segment between nodes represented their regulatory relationship, i.e., activation or inhibition. If a node was not inhibited by any other node, a basal enzyme was assumed to inactivate that node. Specifically, for every topology, nodes A and B received external stimulatory signals X and Y, respectively, and the node C was detected as the output signal. The dynamic behavior of the whole network was determined by the ordinary differential equations of concentrations of all nodes.

For each three-node EN, we used the Latin hypercube sampling method to generate 10,000 parameter sets and independently simulated the input–output response functions. In each simulation, we first determined the concentration range of signal X corresponding to three different concentrations of Y (Y1, Y2, and Y3) within the ratio range of (0.01~10), and then calculated the three response curves (Figure 1A). Next, we evaluated the ratio-sensing performance with a certain parameter set by calculating the RSD between three response curves at sampled X/Y ratios. If and only if the total RSD across all stimulatory ratios was less than 10%, we considered the topological structure with a certain parameter set capable of ratio sensing. We then quantified the robustness for each network topology by the Q-value, which was defined as the fraction of parameter sets with which the topology exhibited ratio-sensing behavior **(**Figure 1B). Obviously, the higher the Q-value was, the more likely it was for the topology to display ratio-sensing behavior, and the less sensitive the behavior was toward parameter perturbations. Topologies with Q-value > 0.001 were regarded viable [27].

### 3.2. Structural Characteristics of Ratio-Sensing Topologies for Enzymatic Networks

In the whole search space of 17,496 enumerated three-node ENs, there were only 1828 networks (~9.29% of the entire topological space) capable of ratio sensing with at least one random parameter combination (Figure S1). Highly robust ratio-sensing networks displayed intermediate regulatory complexity with 2–6 regulatory edges (Figure S2), suggesting that the robust ratio-sensing behavior was significantly dependent on the network topological structure.

Next, we performed topology pruning to remove redundant edges and found the minimal core topologies that exhibited robust ratio sensing. According to previous work [30,36], the minimal core topology was defined as a topology in which each regulatory edge was necessary for the Q-value staying above the 0.001 cutoff, i.e., removing any edge from the topology would result in a Q-value below the 0.001 cutoff. Among all 1828 networks, we found seven minimal ratio-sensing core topologies (Figure 1C). Of the seven core topologies, three (Topo_8383, 7627, and 10567) were significantly higher in robustness (Q-value 0.004–0.035) and had only two edges. The other four networks displayed marginal robustness (Q-value ~0.001) and they shared a more complex network structure consisting of an activation edge from node A to B, an inhibition edge from node B to C, and self-activation of node C. Adding more regulatory edges to the last four network topologies did not improve their robustness, indicating that the topological structure capable of ratio sensing with high robustness was highly constrained within the topological space. Therefore, we further analyzed the essential structural characteristics of highly robust topological structures (Q ≥ 0.001).

Of the 1828 networks capable of ratio sensing, only 374 network topologies satisfied the Q ≥ 0.001 condition. We performed clustering of these 374 highly robust network topologies (Figure 2A). Each row in the cluster diagram represented a highly robust network topology, and each column represented the regulatory relationship (activating, inhibiting, or non-interacting) between a pair of nodes. Clustering analysis revealed four classes of core ratio-sensing motifs according to nodes interactions. Network topologies with the same motif shared a common regulatory structure, named as motifs A, B, C, and D, respectively. These motifs were shown in Figure 2A with their median of robustness rank of motifs. Motifs A, B, and C were generative to most topologies in the high robustness set of 374 networks, while only 10 network topologies were associated to motif D (Figure S3). Comparing the robustness distributions among the four classes of topologies as defined by the core motifs, we found the classes of motifs A, B, and C had higher robustness than the class of motif D (Figure 2B, left). Furthermore, we found that the seven minimal ratio-sensing core topologies fell into the four motif classes. Topo_8383, 7627, and 10567 belonged to motifs A, B, and C, respectively, which had overall high robustness, while the remaining four minimal core topologies belonged to motif D. The consistency in robustness between the seven minimal core topologies and the four motifs confirmed that minimal ratio-sensing core structures played a dominant role in determining the robustness of network topological structures.

### 3.3. Robustness and Evolutionary Connectivity

To investigate the mechanisms of ratio sensing, we derived the mathematical expression of ratio sensing for Topo_8383. This topological structure was consistent with that for the yeast galactose pathway in previous studies [5,6]. The following system of ordinary differential equations modeled the process that stimulatory signals X and Y transformed protein A and B from inactive states to active states through enzymatic regulations, respectively, followed by the enzymatic interactions between proteins A, B, and C and their basal enzymes. Because nodes A and B were not inhibited by any other node, basal enzymes *F_A_* and *F_B_* were assumed to inactivate nodes A and B from their active to inactive form with the Michaelis–Menten constants $K_{{MF}_{A}}$ and $K_{{MF}_{B}}$, respectively,

(1) $$\{\begin{array}{c} \frac{dA}{dt}=S_{x}k_{x}\frac{1-A}{1-A+K_{Mx}}-F_{A}k_{F_{A}}\frac{A}{A+K_{MF_{A}}};\\ \frac{dB}{dt}=S_{y}k_{y}\frac{1-B}{1-B+K_{My}}-F_{B}k_{F_{B}}\frac{B}{B+K_{MF_{B}}};\\ \frac{dC}{dt}=Ak_{A}\frac{1-C}{1-C+K_{MA}}-Bk_{B}\frac{C}{C+K_{MB}}; \end{array}$$

where $K_{Mi}$ (*i* = *A*, *B*, *x* or *y*) and *k_i_* (*i* = *A*, *B*, *x*, *y*, $F_{A}$ or $F_{B}$) were the Michaelis–Menten constants and catalytic rate constants of the enzymes, respectively. In the ordinary differential equations, the total concentrations of each node were normalized to 1. *A*, *B*, and *C* represented the concentration of active states while 1 − *A*, 1 − *B*, and 1 − *C* represented the concentration of the inactive state, which varied from 0 to 1. When $1-A\ll K_{Mx}\;\mathrm{and}\;A\ll K_{MF_{A}}$, let A*, B*, and C* be the steady state concentrations of active A, B, and C, respectively. At steady state, we had

$$A^{*}=\frac{S_{x}}{S_{x}+F_{A}\frac{k_{F_{A}}\cdot K_{Mx}}{k_{x}\cdot K_{MF_{A}}}}=\frac{S_{x}}{S_{x}+\tilde{k_{A}}}\;,\;\;\tilde{k_{A}}=F_{A}\frac{k_{F_{A}}\cdot K_{Mx}}{k_{x}\cdot K_{MF_{A}}}$$

(2) $$B^{*}=\frac{S_{y}}{S_{y}+F_{B}\frac{k_{F_{B}}\cdot K_{My}}{k_{y}\cdot K_{MF_{B}}}}=\frac{S_{y}}{S_{y}+\tilde{k_{B}}}\;,\;\;\tilde{k_{B}}=F_{B}\frac{k_{F_{B}}\cdot K_{My}}{k_{y}\cdot K_{MF_{B}}}$$

$$C^{*}=\frac{Ak_{A}K_{MB}}{Ak_{A}K_{MB}+Bk_{B}K_{MA}}=\frac{S_{x}+\frac{S_{x}}{S_{y}}\tilde{k_{B}}}{\left(1+\tilde{k_{C}}\right)S_{x}+\frac{S_{x}}{S_{y}}\tilde{k_{B}}+\tilde{k_{A}\cdot }\tilde{k_{C}}}\;,\;\;\tilde{k_{C}}=\frac{k_{B}\cdot K_{MA}}{k_{A}\cdot K_{MB}}$$

while

(3) $$\{\begin{array}{c} \frac{S_{x}}{\tilde{k_{B}}}\ll \frac{S_{x}}{S_{y}}\\ \left(1+\tilde{k_{C}}\right)\frac{S_{x}}{\tilde{k_{B}}}\ll \frac{S_{x}}{S_{y}}\\ \frac{\tilde{k_{A}\cdot }\tilde{k_{C}}}{\tilde{k_{B}}}\gg \left(1+\tilde{k_{C}}\right)\frac{S_{x}}{\tilde{k_{B}}} \end{array}$$

(4) $$C^{*}=\frac{S_{x}+\frac{S_{x}}{S_{y}}\tilde{k_{B}}}{\left(1+\tilde{k_{C}}\right)S_{x}+\frac{S_{x}}{S_{y}}\tilde{k_{B}}+\tilde{k_{A}\cdot }\tilde{k_{C}}}\approx \frac{\frac{S_{x}}{S_{y}}}{\frac{S_{x}}{S_{y}}+\frac{\tilde{k_{A}\cdot }\tilde{k_{C}}}{\tilde{k_{B}}}}=f\left(\frac{S_{x}}{S_{y}}\right)$$

The first two conditions of (3) meant $B^{*}\approx \frac{S_{y}}{\tilde{k_{B}}}\ll 1$ and the last one inferred $A^{*}\approx \frac{S_{x}}{\tilde{k_{A}}}$. The result of derivation showed that signal X and Y achieved competitive activation of node C through the node A and B, and thereby led to the ratio-sensing behavior at the output of C under the condition that $S_{y}\ll \tilde{k_{B}}$ and $S_{x}\ll \tilde{k_{A}}$. This conclusion implied that when the network was very sensitive to the dual stimulatory signals X and Y, the output at node C was essentially approximated to a Hill function of the ratio X/Y as mediated by the competitive topology.

A network with high robustness usually combined several network topological motifs. Considering that the overall robustness of motifs A, B, and C were significantly higher than that of the motif D, we explored the dependence of the robustness of combined motifs on the robustness of its constituent motifs A, B, and C (Figure 2B, right). If a network topological structure was a combination of the motifs A and B, it was classified as motif AB. All topologies of class BC naturally contained motif A and were thereby defined as class ABC. We found that network topologies containing motif A had the potential to be more robust regardless of what other motifs were combined. In particular, combining A with C (class AC) slightly enhanced the overall robustness of class A, despite class C ranked lowest in robustness among the three core motifs. The results of motif combination showed that the highly robust network structures could be obtained by the combination of robust motifs. Therefore, we investigated the transformation between motif classes through topological change.

In Figure 2C, all network topologies with Q > 0.0001 were displayed in a metagraph where each node represented a topology, and a link was drawn if two topologies could interconvert through a change in one edge. Each motif class occupied a specific domain and the domains of motif classes A, B and C had a certain overlap (e.g., motif classes AB, ABC, etc.). By calculating the network connectivity within and between different motif classes, we found that motif D was relatively isolated from the other motifs. Natural populations evolving a functional motif proceed in the metagraph through paths defined by interconnected topologies. The robustness of intermediate topologies along their evolutionary paths governs the stability of the path and the accessibility of evolved topologies. Therefore, we applied a robustness threshold to gradually remove non-robust topologies from the metagraph. For high robustness cutoffs, only motif classes A, B, and C were connected with each other in the metagraph, with a higher connectivity between A and C (Figure 2D). It was difficult to find a path with high Q-values from motif class D towards motif classes A, B, and C or vice versa, but it was much more convenient to evolve within motif classes A, B, and C.

### 3.4. Structural Characteristics of Ratio-Sensing Topologies for Transcriptional Networks

While the above analyses focused on three-node ENs, transcriptional regulatory networks (TRNs) accounts for a significant portion of synthetic biological circuits. Therefore, we also enumerated and analyzed the structural space for three-node TRNs to find appropriate network structures for constructing ratio-sensing circuits.

Among all three-node TRNs, there were 1874 (~10.7%) topological structures achieving ratio-sensing behavior (Figures S4–S6). Similarly, the distribution of Q-values indicated that the network topological structure was important for the robust ratio-sensing (Figure S4). Three minimal core topologies, Topo_8383, 7627, and 10567, were extracted for the TRN model (Figure 3A), which were the same three most robust minimal ratio-sensing core topologies for the EN. Although the robustness of Topo_8383 roughly remained unchanged, the robustness of Topo_7627 and Topo_10567 decreased from 0.035 and 0.0136 for EN to 0.0008 and 0.0014 for TRN.

Then, 384 robust topological structures with Q-values over 0.001 were clustered and these highly robust networks were also classified into motifs A, B, and C (Figure 3B), whose structures were identical to motifs A, B, and C for EN. Motif A contained 346 topological structures and had a higher overall robustness for TRN (Figure 3C). The metagraphs exhibited similar characteristics as in EN, with motif classes A, B, and C highly interconnected, indicating topologies could evolve gradually toward robustness (Figure 3D,E).

Similarly, we derived an analytic solution for the output behavior of Topo_8383 from motif A (SI). Under the condition that $K_{A}\ll A^{*}$ and $K_{B}\ll B^{*}$ i.e., when the TRN was very sensitive to the dual stimulatory signals X and Y and $A^{*}$ and $B^{*}$ were saturated in comparison with their binding affinities ($K_{A}$ and $K_{B}$) to the promoter of *C*, the TRN Topo_8383 exhibited robust ratio-sensing behavior. We also simulated the ratio sensing behavior for Topo_8383 transcription networks. The simulation results were shown in Figure S7 with parameters listed in Table S1 in the SI. The ratio sensing of motifs B and C were also analyzed and qualitatively demonstrated (SI, method). Compared to the case of motif A, ratio sensing imposed additional constraints in the Hill coefficients of transcriptional regulation for motifs B and C. These additional constrains might be a reason for the differences in robustness between motifs.

## 4. Discussion

In this work, we enumerated and analyzed the topological characteristics of the ratio-sensing for the three-node ENs and TRNs, respectively. For ENs, we found seven minimal ratio-sensing core topologies and four network topological motifs. The first three most robust core topologies belonged to the first three most robust motifs, respectively, and all of the last four minimal ratio-sensing core topologies belonged to motif D. Among the four motifs, the first three motifs contained most of the highly robust topological structures, and their overall robustness was significantly higher than that of the motif D. The evolutionary space of the networks confirmed that it was difficult to find a path with high Q-value from motif D toward motifs A, B, or C while the evolutionary paths between motifs A, B, and C were much more connected. For TRNs, we found the same motifs A, B, and C and their associated minimal core topologies. Motif D was not robust for TRNs. Consistently, motifs A, B, and C were highly connected in the network evolutionary space.

Our results indicated that the topological characteristics for ratio sensing were revealed through the coarse-grained computational enumeration. Although there were four additional minimal core topologies and a motif D for ENs, motif class D had few connections with motif classes A, B, and C in the evolutionary space. The high cluster degree and the large interconnected topological domains were characteristic of the evolutionary space structure for robust biological networks [39].

**Figure 4:**
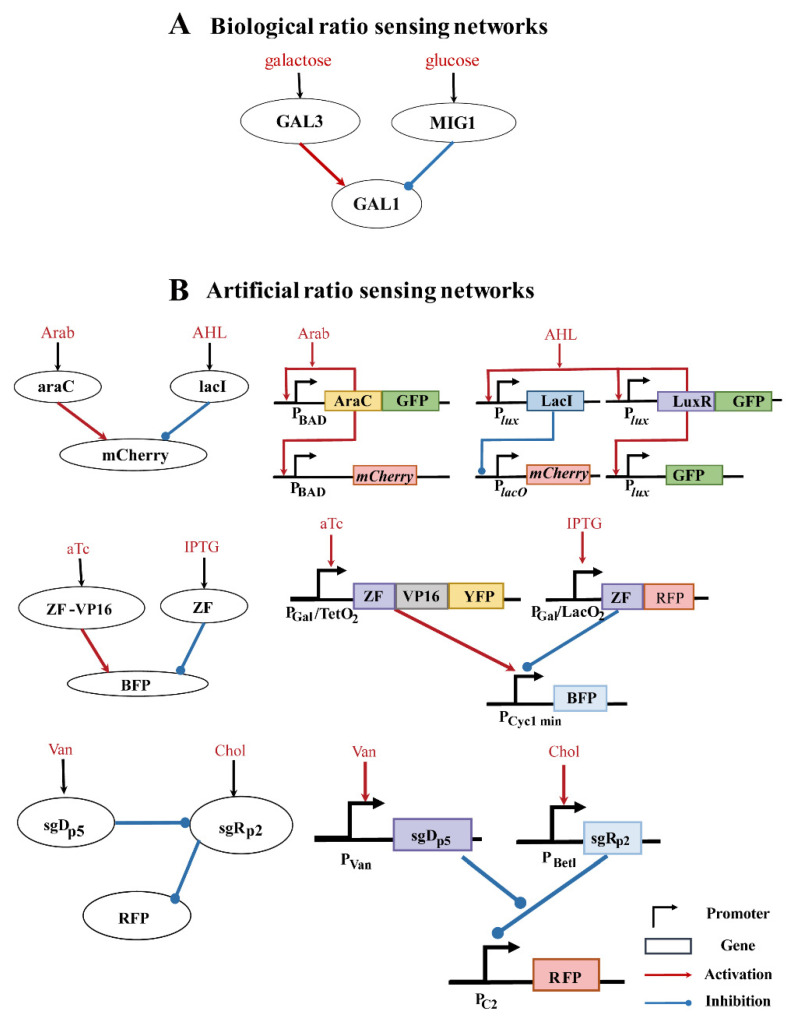
Examples of ratio sensing in natural and synthetic systems. (**A**) Example of a natural ratio sensing in the yeast galactose pathway; (**B**) Examples of synthetic ratio sensing as summarized from references [14,15,16]. Arab, arabinose; AHL, acyl-homoserine lactone; AraC, the AraC protein; LacI, the repressor LacI protein; LuxR, the LuxR protein; GFP, the green fluorescent protein; mCherry, the red fluorescent protein; aTc, anhydrotetracycline; IPTG, isopropyl β-D-1-thiogalactopyranoside; ZF, the Zinc finger protein; VP16, a kind of transcriptional activating domain; BFP, YFP, and RFP are the blue, yellow, and red fluorescent protein, respectively; sgRP2 and sgDP5, sgRNAs guiding the dCas9 repressor protein.

Among the three ratio-sensing motifs, motif A ranked highest in robustness. In fact, many natural regulatory networks capable of ratio sensing adopted motif-A-associated Topo_8383. For example, although the carbon catabolite repression has been widely accepted since 1940s, experiments verified that the glucose metabolism pathway tended to perform ratio-sensing of the two sugar concentrations in yeast (Figure 4A). In this metabolic pathway, glucose and galactose activate genes GAL3 and MIG1, respectively. GAL3 then activates the gene GAL1 whereas MIG1 inhibits GAL1, achieving competitive regulation and a ratio sensing output at GAL1. In addition, the BMP-signaling pathway could generate a ratio-sensing response through differential ligand-receptor affinities by which the weak affinity ligand was “inhibited” by the strong affinity ligand. Some synthetic regulatory circuits of Topo_8383 were constructed in *E. coli* and yeast cells to perform ratio-sensing as well (Figure 4B). In one circuit, the two input signals, arabinose and acyl-homoserine lactone, activated araC and lacI, respectively, which further activated and inhibited the expression of the output signal mCherry [14]. In another circuit, the output signal BFP was activated and inhibited by anhydrotetracycline and IPTG signals, respectively, as mediated by zinc finger transcriptional regulators [15]. Recently, the dCas9 transcriptional repressor was used to constructed a ratio-sensing circuit [16]. Instead of Topo_8383, the circuit adopted Topo_7627. Vanillic acid induced SgDp5 sgRNA expression inhibited the expression of the sgRp2 sgRNA via dCas9. While sgRp2 inhibited the output signal RFP expression via dCas9, it was activated by a second input signal, choline (Figure 4B).

Although synthetic biology had constructed some ratio-sensing circuits such as Topo_7627 by the bottom-up design approach, the robustness of circuits, which could limit the design and implementation of more complex regulatory circuits, should be taken into account for rationally designing ratio-sensing systems. Because of the consistency between the idea of biological reverse engineering and the design process of synthetic biology, we were enlightened to resolve the problem of rationally designing genetic circuits with complex pre-defined functions with the help of reverse engineering. In fact, the methods of reverse engineering included network enumeration, sub-network combination and the Boolean network model. However, sub-network combinations and Boolean network models were more appropriative for large-scale networks. In addition, evolutionary-inspired approaches such as genetic algorithms could find some structures of the target function, but the topologies obtained usually were the local rather than the global optimal solution in the entire topological space. Thus, the network enumeration strategy was suitable for providing robust design schemes for synthetic biology.

These synthetic TRNs imply that our computational results could provide design schemes for synthetic biology to construct high robustness and low complexity networks with ratio-sensing behavior. By taking a systematic approach, we were able to dissect the relationship between network structure and function for a fundamental biological process—ratio sensing. It is conceivable that the successful implementation of functional synthetic circuits would benefit from such reverse engineering approaches which reveal the core design principle through global topological enumeration and analyses.

## Data Availability

Data are included in the article or Supplementary Materials.

## Supplementary Materials

The following supporting information can be downloaded at: https://www.mdpi.com/article/10.3390/life13020351/s1, Figure S1: The distribution of Q-values of enumerated three-node ENs; Figure S2: The distribution of Q-values and the topological complexity of ENs; Figure S3: The structural characteristics and robustness distributions of the four motif classes for ENs with Q-value > 0.0001; Figure S4: The distribution of Q-values of enumerated three-node TRNs; Figure S5: The distribution of Q-values and the topological complexity of TRN; Figure S6: The structural characteristics and robustness distributions of the three motif classes for TRNs with Q-value > 0.0001; Figure S7: An example of ratio-sensing network simulation for the TRN Topo_8383; Table S1: The parameters of the model simulation in Figure S7.
