# Supplementary material for: The Topological Characteristics of Biological Ratio-Sensing Networks
Source: Life (Basel). 2023 Jan 28;13(2):351. doi: 10.3390/life13020351 (PMC9965423; doi:10.3390/life13020351)
Supplement: Supplementary file 1 [file life-13-00351-s001.zip › life-2154664-supplementary.pdf]

Supplementary information for:

The topological characteristics of biological ratio-sensing networks

Xinmao Chen[1], Tianze Wang[2], Ying Guan[3], Qi Ouyang[1,2*], Chunbo Lou[3*], Long Qian[2*]
[1]School of Physics, Peking University, Beijing 100871, China
[2]Center for Quantitative Biology, Peking University, Beijing 100871, China
[3]Center for Cell and Gene Circuit Design, CAS Key Laboratory of Quantitative Engineering Biology, Guangdong Provincial Key Laboratory of Synthetic Genomics, Shenzhen Key Laboratory of Synthetic Genomics, Shenzhen Institute of Synthetic Biology, Shenzhen Institutes of Advanced Technology, Chinese Academy of Sciences, Shenzhen, 518055, China

[*]Correspondence should be addressed to qi@pku.edu.cn (Q.O.), cb.lou@siat.ac.cn (C.L.) and long.qian@pku.edu.cn (L.Q.)

1. Supplementary notes
2. Supplementary figures
3. Supplementary table

1. Supplementary notes

1.1 Robustness of motifs for enzymatic networks and transcriptional networks

In the whole search space of 17496 enumerated three-nodes ENs, there were only 1,828 networks (~ 9.29% of the entire topological space) capable of ratio-sensing with at least one random parameter combination. The distribution of Q-values in Figure S1 showed an exponential downward trend, indicating that the robust ratio-sensing behavior was significantly dependent on the topological structure. The distribution of Q-values and the topological complexity of ENs in Figure S2 indicated that only a few topologies was robust with high Q-values and these topological complexities were mainly concentrated on 2-6 regulatory edges, suggesting that the robust ratio-sensing behavior was significantly dependent on the network's topological structure. Figure S3 showed the robustness difference among the four classes of topologies with their cluster diagram. Obviously, motifs A, B and C had a good overall robustness with intermediate topological complexity, while the highest robustness of motif D was just 0.0019 for the network topological ID Topo_14089.

For enumeration of the three-nodes TRNs, there were 1,874 topologies (~10.7% of the entire topological space) achieving ratio-sensing behavior with at least one random parameter combination. Similar to the above results, details about the distribution of Q value and the complexity were shown in Figure S4~ S6.

1.2 Mathematical analysis of ratio-sensing core topologies

For the ratio-sensing core TRN topology Topo_8383 from motif A, the following system of ordinary differential equations modeled the process that the two transcription factors A and B which regulated the output C production were activated by the stimulatory signals X and Y, respectively.

$$\begin{cases} \dfrac{dA}{dt} = \alpha_A \dfrac{(\frac{S_x}{K_x})^{n_x}}{1 + (\frac{S_x}{K_x})^{n_x}} - \gamma A; \\ \dfrac{dB}{dt} = \alpha_B \dfrac{(\frac{S_y}{K_y})^{n_y}}{1 + (\frac{S_y}{K_y})^{n_y}} - \gamma B; \\ \dfrac{dC}{dt} = \alpha_C \dfrac{(\frac{A}{K_A})^{n_A}}{1 + (\frac{A}{K_A})^{n_A} + (\frac{B}{K_B})^{n_B}} - \gamma C; \end{cases}$$

where K_x, K_y, K_A and K_B were the binding affinities of S_x, S_y, A and B to their operators, respectively, α_A, α_B and α_C were the maximal transcriptional rates, and γ was the rate of degradation and dilution. Let A*, B* and C* be the steady state concentration of A, B and C, respectively. When $(\frac{S_x}{K_x})^{n_x} \ll 1$ and $(\frac{S_y}{K_y})^{n_y} \ll 1$, we had

$$A^* = \frac{\alpha_A}{\gamma} \frac{(\frac{S_x}{K_x})^{n_x}}{1 + (\frac{S_x}{K_x})^{n_x}} \approx \frac{\alpha_A}{\gamma} (\frac{S_x}{K_x})^{n_x}$$

$$B^* = \frac{\alpha_B}{\gamma} \frac{(\frac{S_y}{K_y})^{n_y}}{1 + (\frac{S_y}{K_y})^{n_y}} \approx \frac{\alpha_B}{\gamma} (\frac{S_y}{K_y})^{n_y}$$

$$C^* = \frac{\alpha_C}{\gamma} \frac{\left(\frac{A}{K_A}\right)^{n_A}}{1 + \left(\frac{A}{K_A}\right)^{n_A} + \left(\frac{B}{K_B}\right)^{n_B}}$$

Set $n_A = n_B = n, \quad n_x = n_y = N$ and while $\begin{cases} \left(\frac{\gamma K_A}{\alpha_A}\right)^n \left(\frac{K_x}{S_y}\right)^{nN} \ll \left(\frac{S_x}{S_y}\right)^{nN} \\ \left(\frac{\gamma K_A}{\alpha_A}\right)^n \left(\frac{K_x}{S_y}\right)^{nN} \ll \left(\frac{\alpha_B}{\alpha_A}\right)^n \left(\frac{K_A}{K_B}\right)^n \left(\frac{K_x}{K_y}\right)^{nN} \end{cases}$,

namely $\begin{cases} K_A \ll A^* \\ K_B \ll B^* \end{cases}$

$$C^* = \frac{\alpha_C}{\gamma} \frac{\left(\frac{S_x}{S_y}\right)^{nN}}{\left(\frac{\gamma K_A}{\alpha_A}\right)^n \left(\frac{K_x}{S_y}\right)^{nN} + \left(\frac{S_x}{S_y}\right)^{nN} + \left(\frac{\alpha_B}{\alpha_A}\right)^n \left(\frac{K_A}{K_B}\right)^n \left(\frac{K_x}{K_y}\right)^{nN}} \approx \frac{\alpha_C}{\gamma} \frac{\left(\frac{S_x}{S_y}\right)^{\tilde{n}}}{\left(\frac{S_x}{S_y}\right)^{\tilde{n}} + \tilde{K}^{\tilde{n}}} = F\left(\frac{S_x}{S_y}\right)$$

$$(\tilde{n} = nN)$$

From the mathematical expression, to achieve ratio sensing, a constraint was on the range of the Hill coefficient which was $n_A = n_B$ and $n_x = n_y$. Besides, under the condition that $K_A \ll A^*$ and $K_B \ll B^*$, i.e., when the network was very sensitive to the dual stimulatory signals X and Y and A^* and B^* were saturated in comparison with their binding affinities (K_A and K_B) to the

promoter of C, the TRN Topo_8383 network exhibited robust ratio-sensing behavior. We simulated the ratio sensing behavior for Topo_8383 transcription networks. This is the topology representative of the Galactose network and the two synthetic ratio-sensing networks in Figure 4. The simulation results were shown in Figure S7 with parameters listed in Table S1.

For the ratio-sensing core topology Topo_7627 from motif B, we had

$$\begin{cases} \dfrac{dA}{dt} = \alpha_A \dfrac{(\frac{S_x}{K_x})^{n_x}}{1+(\frac{S_x}{K_x})^{n_x}} - \gamma A; \\ \dfrac{dB}{dt} = \alpha_B \dfrac{(\frac{S_y}{K_y})^{n_y}}{1+(\frac{S_y}{K_y})^{n_y}} \dfrac{(\frac{A}{K_A})^{n_A}}{1+(\frac{A}{K_A})^{n_A}} - \gamma B; \\ \dfrac{dC}{dt} = \alpha_C \dfrac{1}{1+(\frac{B}{K_B})^{n_B}} - \gamma C; \end{cases}$$

When $(\frac{S_x}{K_x})^{n_x} \ll 1$ and $(\frac{S_y}{K_y})^{n_y} \ll 1$,

$$A^* = \frac{\alpha_A}{\gamma} \frac{(\frac{S_x}{K_x})^{n_x}}{1+(\frac{S_x}{K_x})^{n_x}} \approx \frac{\alpha_A}{\gamma}(\frac{S_x}{K_x})^{n_x}$$

$$B^* = \frac{\alpha_B}{\gamma} \frac{(\frac{S_y}{K_y})^{n_y}}{1+(\frac{S_y}{K_y})^{n_y}} \frac{1}{1+(\frac{A}{K_A})^{n_A}} \approx \frac{\alpha_B}{\gamma} \frac{(\frac{S_y}{K_y})^{n_y}}{1+(\frac{A}{K_A})^{n_A}}$$

$$C^* = \frac{\alpha_C}{\gamma} \frac{1}{1+\left(\frac{B}{K_B}\right)^{n_B}}$$

Set $n_A = n_B = 1,\ n_x = n_y = N$ and while $\begin{cases} \frac{\gamma K_A}{\alpha_A}\left(\frac{K_x}{S_y}\right)^N \ll \left(\frac{S_x}{S_y}\right)^N \\ \frac{\gamma K_A}{\alpha_A}\left(\frac{K_x}{S_y}\right)^N \ll \frac{\alpha_B}{\alpha_A}\left(\frac{K_x}{K_B K_y}\right)^N \end{cases}$, namely $\begin{cases} K_A \ll A^* \\ K_B \ll B^* \end{cases}$

$$C^* = \frac{\alpha_C}{\gamma} \frac{\frac{\gamma K_A}{\alpha_A}\left(\frac{K_x}{S_y}\right)^N + \left(\frac{S_x}{S_y}\right)^N}{\frac{\gamma K_A}{\alpha_A}\left(\frac{K_x}{S_y}\right)^N + \left(\frac{S_x}{S_y}\right)^N + \left(\frac{\alpha_B}{\alpha_A}\right)\left(\frac{K_x}{K_B K_y}\right)^N} \approx \frac{\alpha_C}{\gamma} \frac{\left(\frac{S_x}{S_y}\right)^N}{\left(\frac{S_x}{S_y}\right)^N + \widetilde{K}^N} = F\left(\frac{S_x}{S_y}\right)$$

$$\widetilde{K} = \frac{K_x}{K_B K_y}\left(\frac{\alpha_B}{\alpha_A}\right)^{\frac{1}{N}}$$

Similarly, ratio-sensing behavior could be obtained with the condition $n_A = 1, n_B = 2$. Obviously, the constraint on the Hill coefficient was much stronger than that of the Topo_8383 from motif A. It meant the network performed ratio sensing with a narrower parameter range, which was consistent with a smaller Q value. Moreover, under the condition that $K_A \ll A^*$ and $K_B \ll B^*$, the TRN Topo_7627 network could exhibit robust ratio-sensing behavior.

For the ratio-sensing core topology Topo_10567 from motif C, we had

$$
\begin{cases}
\dfrac{dA}{dt} = \alpha_A \dfrac{(\frac{S_x}{K_x})^{n_x}}{1+(\frac{S_x}{K_x})^{n_x}} \dfrac{(\frac{B}{K_B})^{n_B}}{1+(\frac{B}{K_B})^{n_B}} - \gamma A; \\
\dfrac{dB}{dt} = \alpha_B \dfrac{(\frac{S_y}{K_y})^{n_y}}{1+(\frac{S_y}{K_y})^{n_y}} - \gamma B; \\
\dfrac{dC}{dt} = \alpha_C \dfrac{(\frac{A}{K_A})^{n_A}}{1+(\frac{A}{K_A})^{n_A}} - \gamma C;
\end{cases}
$$

When $(\frac{S_x}{K_x})^{n_x} \ll 1$ and $(\frac{S_y}{K_y})^{n_y} \ll 1$,

$$
A^* = \frac{\alpha_A}{\gamma} \frac{(\frac{S_x}{K_x})^{n_x}}{1+(\frac{S_x}{K_x})^{n_x}} \frac{1}{1+\left(\frac{B}{K_B}\right)^{n_B}} \approx \frac{\alpha_A}{\gamma} \frac{(\frac{S_x}{K_x})^{n_x}}{1+\left(\frac{B}{K_B}\right)^{n_B}}
$$

$$
B^* = \frac{\alpha_B}{\gamma} \frac{(\frac{S_y}{K_y})^{n_y}}{1+(\frac{S_y}{K_y})^{n_y}} \approx \frac{\alpha_B}{\gamma} (\frac{S_y}{K_y})^{n_y}
$$

$$
C^* = \frac{\alpha_C}{\gamma} \frac{A^{n_A}}{{K_A}^{n_A} + A^{n_A}}
$$

Set $n_A = n_B = 1,\ n_x = n_y = N$ and while $\begin{cases} \frac{\gamma K_A}{\alpha_A}\left(\frac{K_x}{S_y}\right)^N \ll \left(\frac{S_x}{S_y}\right)^N \\ \frac{\gamma K_A}{\alpha_A}\left(\frac{K_x}{S_y}\right)^N \ll \left(\frac{\alpha_B K_A}{\alpha_A K_B}\right)\left(\frac{K_x}{K_y}\right)^N \end{cases}$, namely $\begin{cases} K_A \ll A^* \\ K_B \ll B^* \end{cases}$

$$
C^* = \frac{\alpha_C}{\gamma} \frac{\left(\frac{S_x}{S_y}\right)^N}{\frac{\gamma K_A}{\alpha_A}\left(\frac{K_x}{S_y}\right)^N + \left(\frac{S_x}{S_y}\right)^N + \left(\frac{\alpha_B K_A}{\alpha_A K_B}\right)\left(\frac{K_x}{K_y}\right)^N} \approx \frac{\alpha_C}{\gamma} \frac{\left(\frac{S_x}{S_y}\right)^N}{\left(\frac{S_x}{S_y}\right)^N + \widetilde{K}^N} = F\left(\frac{S_x}{S_y}\right)
$$

$$
\widetilde{K} = \frac{K_x}{K_y}\left(\frac{\alpha_B K_A}{\alpha_A K_B}\right)^{\frac{1}{N}}
$$

Ratio sensing could be obtained with $n_A = 2, n_B = 1$. Obviously, a stronger Hill coefficient constraint was imposed on Topo_10567, which might be a reason for the differences in robustness between motifs. Furthermore, the condition that $K_A \ll A^*$ and $K_B \ll B^*$ was important for Topo_10567 to ratio sensing.

2. Supplementary figures

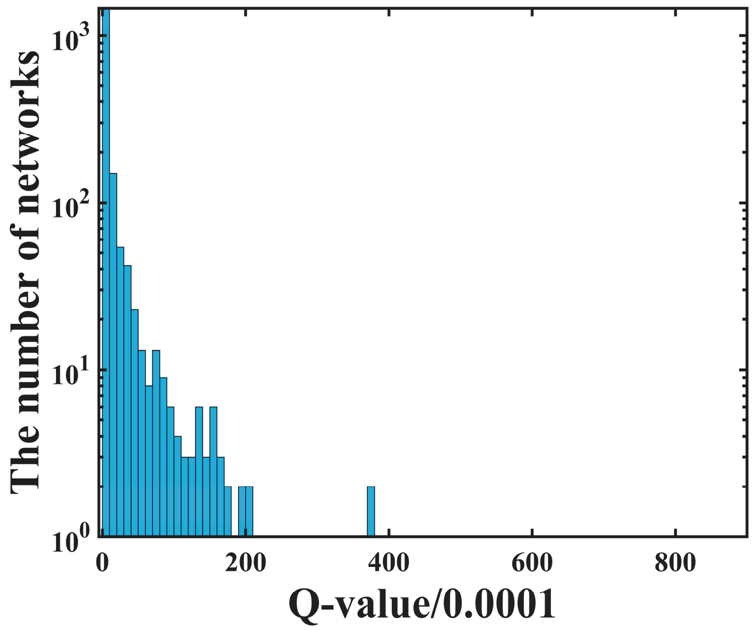

Figure S1: The distribution of Q-values of enumerated three-nodes ENs.

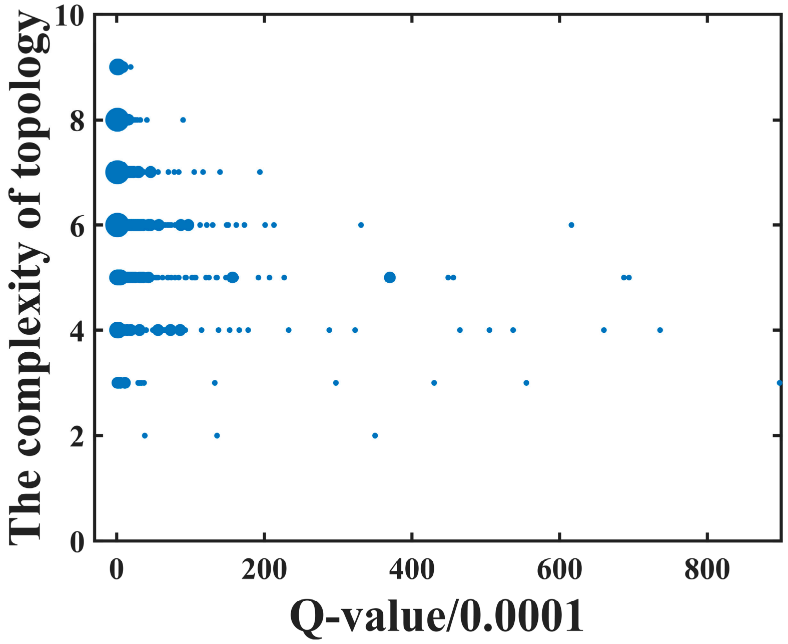

Figure S2: The distribution of Q-values and the topological complexity of ENs. The complexity of each topology was the total number of the regulatory edges between nodes. The circle size was proportional to the number of networks with the complexity and Q.

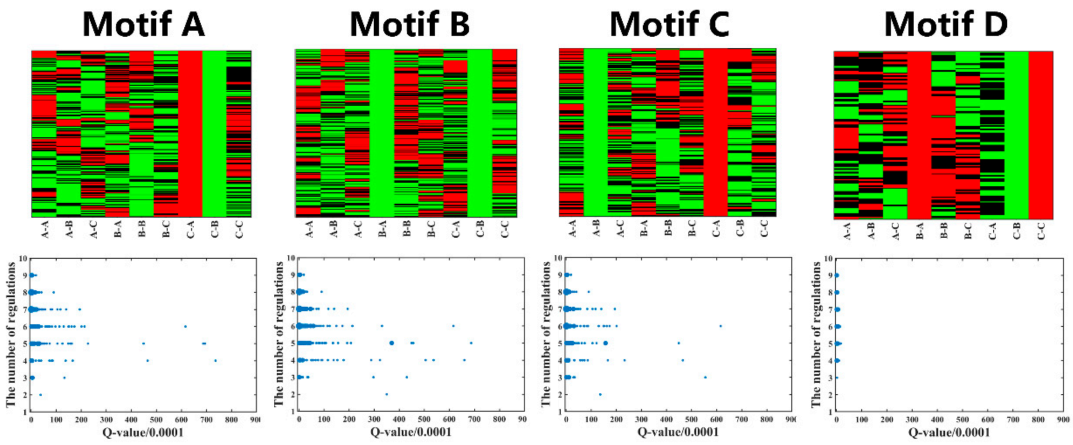

Figure S3: The structural characteristics and robustness distributions of the four motif classes for ENs with Q-value>0.0001. The top panel shows the cluster diagram of the four motif classes. The bottom panel shows the distribution of the topological complexity and robustness. Each column corresponding to the topologies in a motif class.

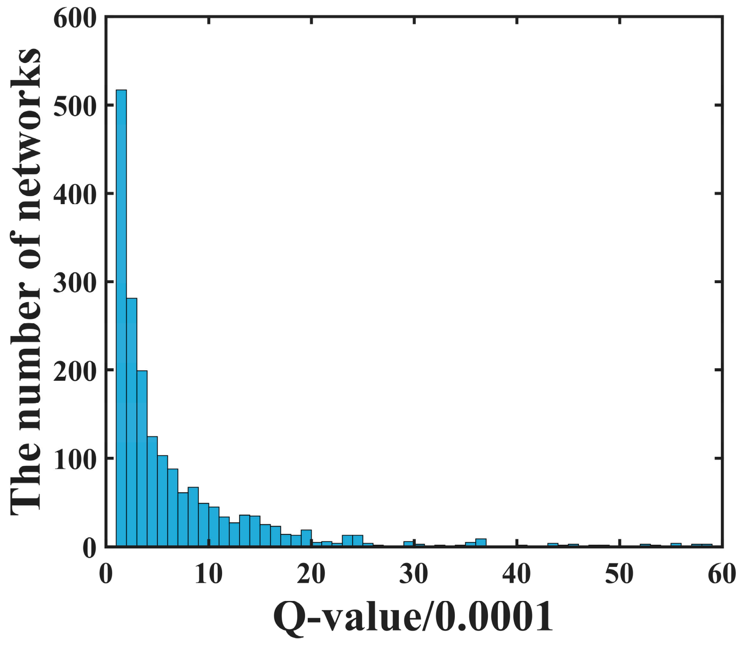

Figure S4: The distribution of Q-values of enumerated three-nodes TRNs.

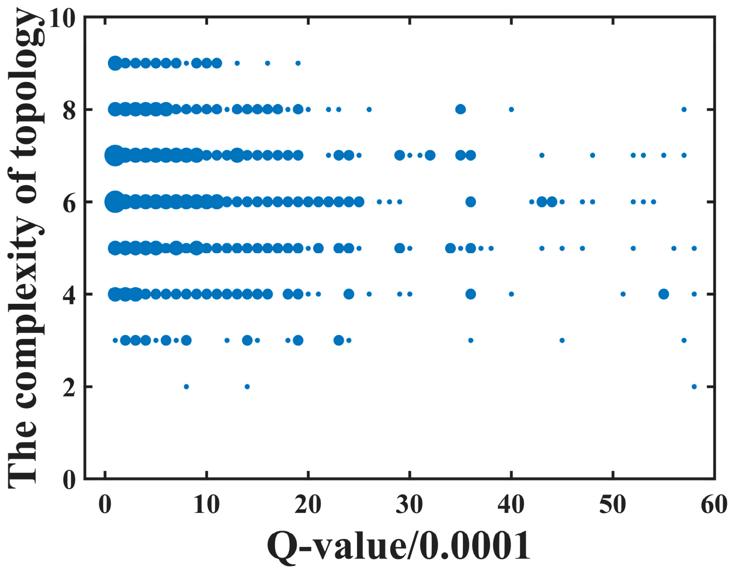

Figure S5: The distribution of Q-values and the topological complexity of TRNs. The complexity of each topology was the total number of the regulatory edges between nodes. The circle size was proportional to the number of networks with the complexity and Q.

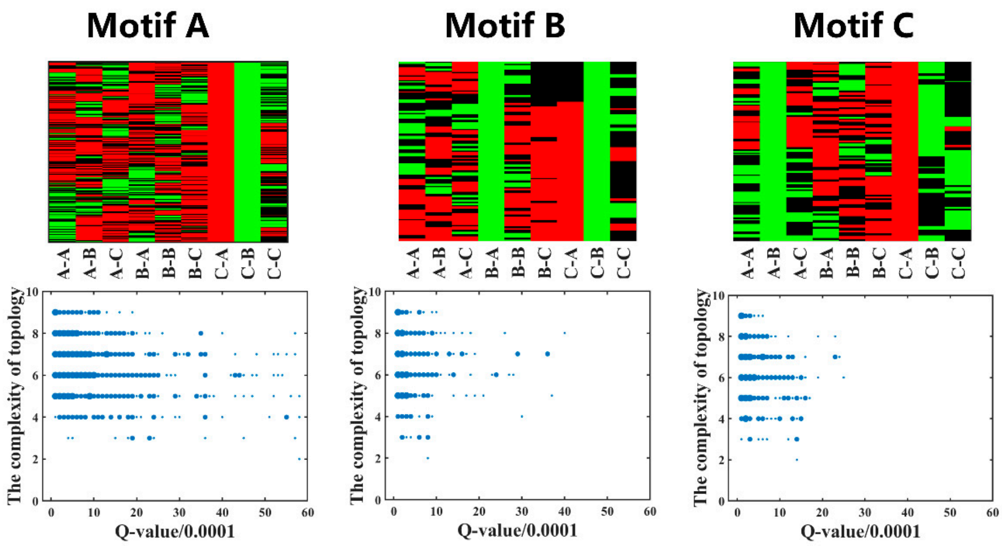

Figure S6: The structural characteristics and robustness distributions of the three motif classes for TRNs with Q-value>0.0001. The top panel shows the cluster diagram of the three motif classes. The bottom panel shows the distribution of the topological complexity and robustness. Each column corresponding to the topologies in a motif class.

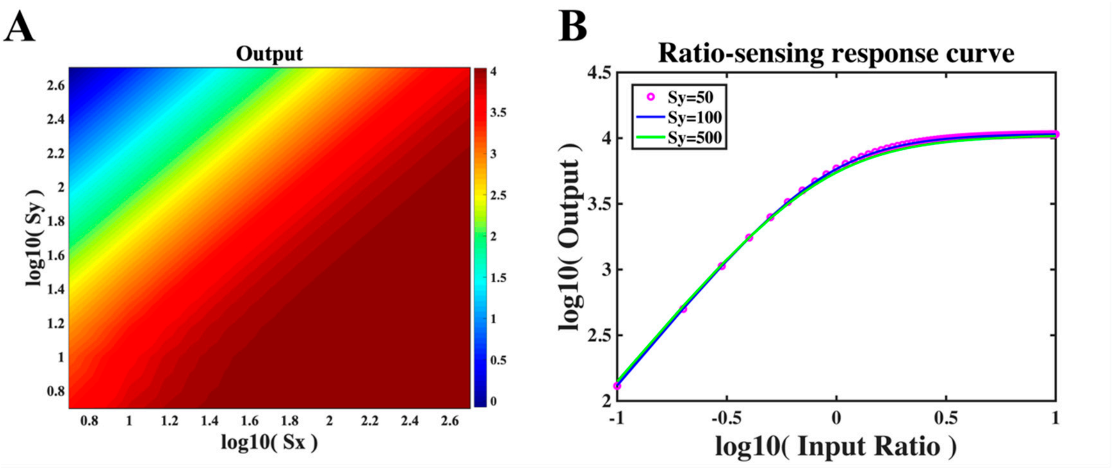

Figure S7: An example of ratio-sensing network simulation for the TRN Topo_8383. The parameters of the model were shown in the following table 1.

3. Supplementary table

Table S1. The parameters of the model simulation in Figure S7.

Parameter	value	Parameter	value	Parameter	value
K_x	3.3997*1e3	α_A	1.569*1e4	n	2
K_y	8.2817*1e3	α_B	2.0880*1e4	N	1
K_A	0.1912	α_C	1.5017*1e4		
K_B	2.0826				
